# Construction of Transport Channels by HNTs@ZIF-67 Composites in a Mixed-Matrix Membrane for He/CH_4_ Separation

**DOI:** 10.3390/membranes15070197

**Published:** 2025-06-30

**Authors:** Jiale Zhang, Huixin Dong, Fei Guo, Huijun Yi, Xiaobin Jiang, Gaohong He, Wu Xiao

**Affiliations:** 1 State Key Laboratory of Fine Chemicals, Frontier Science Center for Smart Materials, Dalian University of Technology, 2 Linggong Road, Dalian 116024, China; zhangjiale@mail.dlut.edu.cn (J.Z.); dong@mail.dlut.edu.cn (H.D.); guofei@mail.dlut.edu.cn (F.G.); yihuijun@mail.dlut.edu.cn (H.Y.); xbjiang@dlut.edu.cn (X.J.); hgaohong@dlut.edu.cn (G.H.); 2 Beijing Yanshan Petrochemical High-Tech Company Limited, Beijing 102500, China

**Keywords:** composite material, HNTs, ZIF-67, mixed matrix membranes, He separation

## Abstract

In this work, HNTs@ZIF-67 composites were synthesized using the in situ growth method and incorporated into 6FDA-TFMB to prepare mixed-matrix membranes (MMMs). Scanning electron microscope (SEM) and transmission electron microscope (TEM) proved that the HNTs@ZIF-67 composite not only retained the hollow structure of HNTs, but also formed a continuous ZIF-67 transport layer on the surface of HNTs. The results of gas permeability experiments showed that with the increase in HNTs@ZIF-67 incorporation, the He permeability and He/CH_4_ selectivity of MMMs showed a trend of increasing first and then decreasing. When the loading is 5 wt%, the He permeability and He/CH_4_ selectivity of MMMs reach 116 Barrer and 305, which are 22.11% and 79.41% higher than the pure 6FDA-TFMB membrane. The results of density functional theory (DFT) and Monte Carlo (MC) calculations reveal that He diffuses more easily inside ZIF-67, HNTs and 6FDA-TFMB than CH_4_, and ZIF-67 shows larger adsorption energy with He than HNTs and 6FDA-TFMB, indicating that He is easily adsorbed by ZIF-67 in MMMs. Based on experimental and molecular simulation results, the mechanism of HNTs@ZIF-67 improving the He/CH_4_ separation performance of MMMs was summarized. With the advantage of a smaller molecular kinetic diameter, He can diffuse through ZIF-67 on the tube orifice of HNTs@ZIF-67 and enter the HNTs’ hollow tube for rapid transmission. At the same time, He can also be rapidly transferred in the continuous ZIF-67 transport channel layer, which improves the He permeability and the He/CH_4_ selectivity of MMMs.

## 1. Introduction

As a non-renewable rare gas, helium is commonly employed in aerospace, medical, and other high-tech fields due to its non-toxicity, non-flammability and low boiling point, which make it an indispensable and irreplaceable strategic resource. According to projections, global helium demand will increase by 6% per year, making helium shortage a key issue in the scientific research field [1,2,3]. However, the reserves of helium on the earth mainly exist in natural gas, making separating helium from natural gas the main way to obtain helium. The main component of natural gas is methane, and the concentration of helium is usually low, at about 1~4 vol% [4]. Especially in China, most natural gas contains helium at a concentration lower than 1 vol%, thus making the separation of helium and methane a key step in helium extraction [1,4]. Therefore, efficient separation technology is needed to extract and purify helium. Membrane separation technology stands out because of its low energy consumption, simple operation, easy coupling and environmental friendliness. Therefore, the development of high-performance He/CH_4_ separation membrane materials has become an important research direction of helium extraction technology [2,4,5,6].

At present, the separation of helium (He) and methane (CH_4_) usually uses polyimide membranes (such as Matrimid 5218) and microporous polymer membranes (such as PIMs) [6,7,8]. However, these polymer structures typically struggle to have high He permeability and high He/CH_4_ selectivity simultaneously. Matrimid 5218 possesses a high degree of chain packing density and has limited chain mobility, which makes its He/CH_4_ selectivity as high as 130, but its helium permeability is only 23 Barrer [8,9]. Although PIMs have large free volumes and high permeability, the large chain mobility also leads to poor long-term stability and low selectivity, which limits their industrial application. It has been reported that the polyimide membranes of 4,4′-(Hexafluoroisopropylidene) diphthalic anhydride(6FDA) and 2,2′-bis(trifluoromethyl) benzidine (TFMB) exhibit excellent He/CH_4_ separation ability. There are a large number of trifluoromethyl (-CF_3_) groups in the side chain of 6FDA-TFMB, which reduces the chain stacking and increases the free volume; both effects synergistically improve the permeability of helium. Moreover -CF_3_ can also inhibit chain rotation and migration, which hinders the diffusion of CH_4_ and enhances the selectivity of He/CH_4_ [7]. In addition, 6FDA-TFMB has good thermal stability and mechanical properties, showing industrial application potential. However, 6FDA-TFMB as a polymer membrane still has a trade-off effect between permeability and selectivity, which limits the further improvement of membrane performance.

By using a mixed-matrix membrane, it is possible to effectively incorporate the dual advantages of matrix and filler, breaking the conventional trade-off between selectivity and permeability of the polymer membranes [10,11,12,13,14]. Therefore, the He/CH_4_ separation performance of 6FDA-TFMB is also expected to be further improved by preparing the MMMs. Thanks to their high porosity, tunable pore structure, and easy chemical modification, metal–organic frameworks (MOFs) are widely used in gas separation [15]. Among many MOFs, ZIF-67 is a three-dimensional porous crystalline material formed by the self-assembly of cobalt ions (Co^2+^) and 2-methylimidazole through coordination bonds, and it is considered to be a potential He/CH_4_ separation material because of its theoretical effective pore size (3.3 Å), which lies between the kinetic diameters of He (2.6 Å) and CH_4_ (3.8 Å) [16]. The addition of ZIF-67 theoretically only contributes to improving the selectivity of the MMMs. An effective method to boost the permeability and selectivity of MMMs concurrently is to use a hollow one-dimensional (1D) tubular material to form a fast transfer channel and serve as a template for growing a dense MOF selection layer outside [17]. Guo et al. [18] reported that UiO-66 was grown on hollow 1D halloysite nanotubes (HNTs) to obtain UiO-66@HNT, and when UiO-66@HNT was introduced into Pebax, it simultaneously enhanced the CO_2_ permeability and CO_2_/N_2_ selectivity of MMMs. Lin et al. [19] used 1D hollow carbon nanotubes (CNTs) as templates and in situ grew ZIF-8 on it, improving the dispersibility of ZIF-8 in MMMs, and enhancing the C_3_H_6_ permeability of and C_3_H_6_/C_3_H_8_ selectivity of MMMs. To further enhance the interfacial compatibility between the filler and the matrix, Lin et al. [20] grew NH_2_-MIL-101 on CNTs, where -NH_2_ forms hydrogen bonds with -CF_3_ in 6FDA-Durene, and the synthesized ZIF-8/CNTs composites not only improved the CO_2_ permeability of MMMs but also enhanced CO_2_/CH_4_ selectivity. Therefore, the design of a filler that grows MOF outside the hollow tube is a practical approach to enhance the permeability and selectivity of MMMs concurrently. As environmentally friendly and low-cost 1D hollow nanotubes, HNTs show hollow structures that can provide transport channels for gas molecules and reduce mass transfer resistance [21,22]. Meanwhile, the surface of HNTs contains abundant -OH active groups, which can provide growth sites for ZIF-67 and contribute to the successful preparation of composite materials. When dense ZIF-67 is grown on the surface of 1D hollow HNTs and encapsulates both ends of the HNT tube orifice, it will not only obtain high He/CH_4_ selectivity but also facilitate He transport through the 1D nano-hollow composite material.

In this work, the HNTs@ZIF-67 composite material was first prepared and characterized by XRD, FTIR, SEM, TEM, and other methods to observe the structure and properties of the composite material. Subsequently, it was incorporated into 6FDA-TFMB to prepare the HNTs@ZIF-67/6FDA-TFMB membrane. Then, the effects on the He/CH_4_ separation performance of MMMs were investigated. These effects were caused by differences in loading, temperature, and pressure. Finally, the mechanism by which HNTs@ZIF-67 improves the gas separation performance of MMMs was systematically elucidated according to the adsorption density field of He/CH_4_ on the surface of different materials in MMMs, using simulation based on Monte Carlo. It provides a theoretical basis for further research and application of HNTs@ZIF-67-like composites and 6FDA-TFMB in the field of He/CH_4_ separation.

## 2. Materials and Methods

### 2.1. Materials

Cobaltous nitrate hexahydrate (Co(NO_3_)_2_·6H_2_O, AR) was purchased from Xilong Technology Co., Ltd. (Shantou, China). 2-Methylimidazole (C_4_H_6_N_2_, 98%) and Dimethylacetamide (DMAC, 99.5%) were purchased from Aladdin Biochemical Reagent Co., Ltd. (Shanghai, China). Halloystite nanotubes (HNTs, >98%) were purchased from Jina New Materials Technology Co., Ltd. (Foshan, China). Ethanol (AR) was purchased from Damao Chemical Reagent Factory (Tianjin, China). High-purity He and CH_4_ gases were provided by the Dalian Institute of Chemical Physics (Dalian, China). 6FDA-TFMB was purchased from Huaxia Shenzhou New Material Co., Ltd. Arkema (Zibo, China).

### 2.2. Preparation of HNTs@ZIF-67

**Figure 1:**
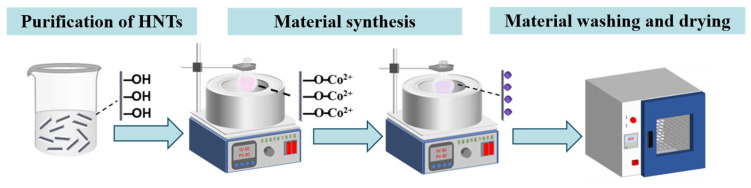
Diagrammatic sketch of the preparation process for HNTs@ZIF-67 composites.

A schematic diagram of the steps for preparing HNTs@ZIF-67 is shown in Figure 1. The synthesis steps of HNTs@ZIF-67 were as follows: (1) Purification of HNTs. An appropriate amount of HNT powder was placed in deionized water and sonicated for 1 h. Then the purified HNTs were separated by centrifugation and placed in an oven at 100 °C for 24 h to obtain dry purified HNTs. (2) Material synthesis. A total of 80 mg of purified HNTs was added to ethanol and sonicated for 30 min to obtain a uniformly dispersed HNT suspension. Then, 1.28 g dimethylimidazole and 0.08 g cobalt nitrate hexahydrate were dispersed in 10 mL ethanol. Next, the HNT suspension was heated, and the dimethylimidazole solution and cobalt nitrate hexahydrate solution were rapidly added until the temperature reached 80 °C; then the mixture was stirred violently for 10 min to obtain an HNTs@ZIF-67 suspension. (3) Material washing and drying—The HNTs@ZIF-67 suspension was centrifuged at 3000 rpm, and the obtained lower layer precipitate was washed with ethanol 3 times. Finally, the washed precipitate was dried under vacuum conditions at 85 °C to obtain HNTs@ZIF-67 powder.

### 2.3. Preparation of HNTs@ZIF-67/6FDA-TFMB MMMs

**Figure 2:**
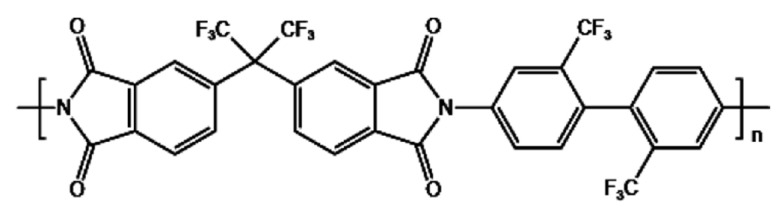
Structure diagram of 6FDA-TFMB [7].

**Figure 3:**
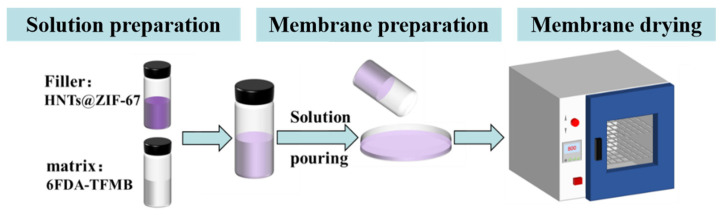
Schematic diagram of the preparation process of HNTs@ZIF-67/6FDA-TFMB MMMs.

The preparation steps of HNTs@ZIF-67/6FDA-TFMB MMMs are as follows (1) Solution preparation—The HNTs@ZIF-67 fillers and matrix 6FDA-TFMB (structure shown in Figure 2) were, respectively, vacuum dried at 85 °C and 150 °C for 12 h to remove excess residual organic solvents and water. Then, 0.2 g 6FDA-TFMB was dissolved in anhydrous DMAC to prepare a 10 wt% solution, and then different proportions of dried HNTs@ZIF-67 were added in anhydrous DMAC and sonicated to achieve uniform dispersion. (2) Membrane preparation. A 10 wt% 6FDA-TFMB solution was poured into an HNTs@ZIF-67 solution and stirred for 24 h to obtain the casting solution. The stirred casting solution was sonicated for 6 h to defoam it, and then the casting solution was decanted into a glass Petri dish. (3) Membrane drying. The Petri dishes with the casting solution were put in an oven at 55 °C for 12 h, 85 °C for 6 h, and 120 °C for 6 h, respectively, and finally, they were put in a vacuum oven at 150 °C for 12 h to obtain MMMs. The Schematic diagram of the preparation process of MMMs is shown in Figure 3.

The loading amount of HNTs@ZIF-67 is calculated by Equation (1).

(1) $$\omega_{HNTs@ZIF\text{-}67}=\frac{m_{HNTs@ZIF\text{-}67}}{m_{HNTs@ZIF\text{-}67}+m_{6FDA\text{-}TFMB}}\times 100\%$$

where $m_{HNTs@ZIF\text{-}67}$ and $m_{6FDA\text{-}TFMB}$ are the mass of HNTs@ZIF-67 and matrix 6FDA-TFMB, respectively.

In this work, MMMs with HNTs@ZIF-67 loadings of 0 wt%, 1 wt%, 3 wt%, 5 wt%, and 7 wt% were prepared.

### 2.4. Characterization of Fillers and Membranes

HNTs@ZIF-67 and MMMs were analyzed by wide-angle X-ray diffraction (XRD) using a Smartlab 9 kW X-ray diffractometer (Akishima, Tokyo, Japan) at the scanning angle within 5–70° with a scanning rate of 2°/min. Fourier transform infrared spectroscopy (FTIR) was recorded using a Bruker Magna 560 spectrometer in the wavenumber range of 4000 to 400 cm^−1^. A Nova Nano SEM 450 scanning electron microscope (SEM) (Billerica, MA, USA) was used to observe the morphology of HNTs@ZIF-67 and MMMs, and a JEM-F200 transmission electron microscope (TEM) (Akishima, Tokyo, Japan) was used to observe the hollow structure of HNTs and HNTs@ZIF-67. The pore structure of HNTs@ZIF-67 was analyzed by a Micromeritics ASAP 2460 Brunner Emmet Teller (BET). The thermal stability of HNTs@ZIF-67 and MMMs was measured by the Q50 TGA. The experiment was carried out at a heating rate of 10 °C/min, and the nitrogen flow rate was maintained from room temperature to 800 °C.

### 2.5. Gas Permeability Test

In this work, a constant volume and variable pressure gas permeation tester was used to evaluate the gas permeability of MMMs. First, an appropriate size of the membrane was cut out and pasted between the copper sheet and the filter paper with glue to obtain the test membrane. After the glue was completely solidified, the test membrane was placed in the membrane pool, and the downstream and upstream pipelines of the membrane pool were pumped to a near-vacuum state. After the upstream and downstream pressure values were stable, N_2_, CH_4_, and He were introduced into the upstream pipeline to test the permeability of a single gas. The change in downstream pressure data during the test time was collected by the pressure sensor. Finally, combined with the thickness and area of the membrane, the permeation rate and selectivity of different gases were calculated under the conditions of 0.2–0.5 MPa and 25–55 °C. The calculation of gas permeability and selectivity is as shown in Equations (2) and (3):

(2) $$P=\frac{VL}{ART\left(p_{1}-p_{2}\right)}(\frac{{dp}_{2}}{dt})$$

(3) $$\alpha_{{He/CH}_{4}}=\frac{P_{He}}{P_{{CH}_{4}}}$$

where *P* is the permeability of gas (Barrer, 1 Barrer = 10^−10^ cm^3^ (STP)·cm·cm^−2^·s^−1^·cmHg^−1^), *L* is the thickness of the membrane (cm), *V* is the volume of the permeate side (cm^3^), *p*_1_ is the feed side pressure, *p*_2_ is the permeate side pressure (cmHg), *A* is the effective membrane area (cm^2^), and *T* is the absolute temperature, $\frac{{dp}_{2}}{dt}$ is the pressure rise rate (cmHg·s^−1^), *R* is the ideal gas constant (0.278 cmHg·cm^3^·cm^−3^ (STP)·K^−1^), $\alpha_{{He/CH}_{4}}$ is the selectivity between methane and helium, $P_{He}$ is the permeability of helium, and $P_{{CH}_{4}}$ is the permeability of methane.

### 2.6. Molecular Simulation

#### 2.6.1. Density Functional Theory Calculation (DFT)

All simulation calculations were performed by Materials Studio (MS) developed by Accelrys. DFT can be used to predict the adsorption sites, adsorption energy, and adsorption configuration of molecules on solid surfaces by calculating the ground state energy and electron density of molecules [23]. DFT calculation can be used to compare the adsorption energy of He and CH_4_ in this research system. In this work, DFT calculation is completed by the DMol^3^ module in Materials Studio.

#### 2.6.2. Monte Carlo Calculation (MC)

Based on molecular dynamics, the interaction between the adsorbate and the surface of the porous material is obtained by the Monte Carlo algorithm [23,24], and then the adsorption isotherm surface, the adsorption energy field, and the adsorption density field can be obtained, to determine the adsorption site of the adsorbate in the material. In this work, the Sorption module is used to obtain the density field of gas molecules in the research system based on the Grand Canonical Monte Carlo [14].

## 3. Results and Discussions

### 3.1. Characterization of HNTs@ZIF-67 Composites

**Figure 4:**
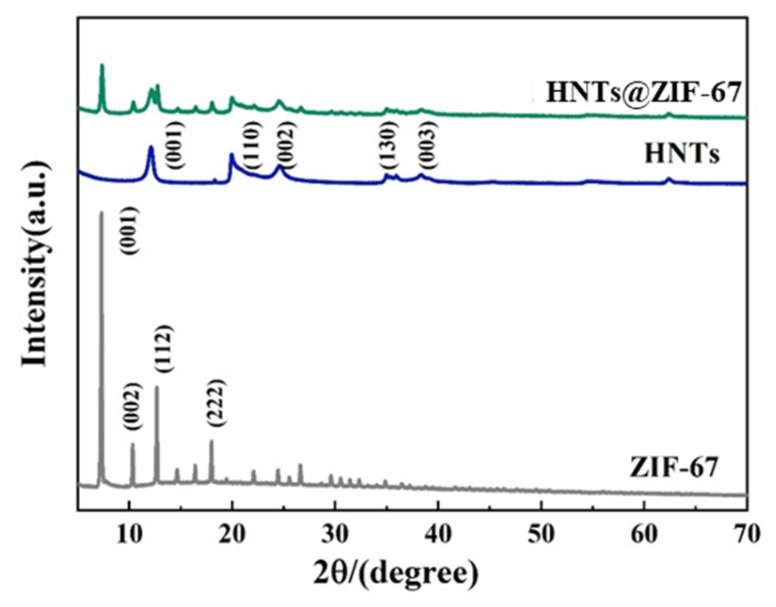
XRD spectra of ZIF-67, HNTs and HNTs@ZIF-67.

The structure of HNTs@ZIF-67 composites was analyzed by X-ray diffraction (XRD). The XRD results of HNTs@ZIF-67 composites are shown in Figure 4. The XRD data of HNTs show diffraction peaks at 12.11°, 20.07°, and 24.57°. These diffraction peaks can also be found in the XRD pattern of HNTs@ZIF-67. The XRD data of ZIF-67 showed sharp diffraction peaks at 2θ = 5.36° and 12.48°, which also appeared in the XRD data of HNTs@ZIF-67. The above results show that the diffraction peaks of HNTs and ZIF-67 appear in HNTs@ZIF-67 at the same time, indicating that ZIF-67 has successfully been introduced into HNTs and grown on them, without destroying their crystal structure.

**Figure 5:**
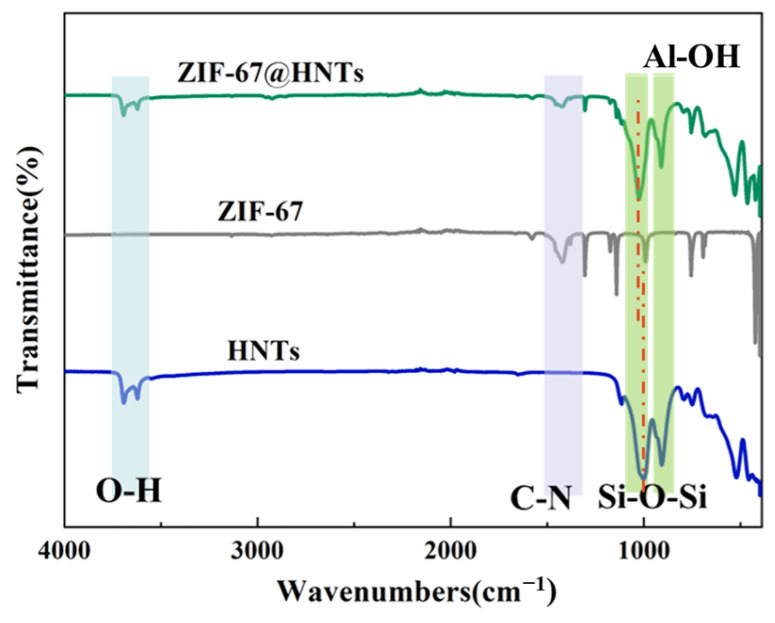
FTIR spectra of ZIF-67, HNTs and HNTs@ZIF67.

Fourier transform infrared spectroscopy (FTIR) was used to explore the molecular structure and chemical composition of the synthesized HNTs@ZIF-67. The FTIR data of ZIF-67, HNTs@ZIF-67 and HNTs are shown in Figure 5. In the FTIR spectrum of HNTs, the peaks at 3610 cm^−1^ and 3640 cm^−1^ corresponded to -OH [25,26]. In addition, the characteristic peaks at 1031 cm^−1^ and 908 cm^−1^ in the original HNT spectrum, were attributed to the tensile vibration of Si–O–Si and the bending vibration of Al-OH, respectively [27]. The peak at 470 cm^−1^ represents the bending vibration of the Si–O bond. The peak at about 1250 cm^−1^ in ZIF-67 is associated with the stretching vibration of the C–N bond in dimethylimidazole [28,29]. The above unique peaks of HNTs and ZIF-67 all appeared in the spectra of HNTs@ZIF-67, indicating that ZIF-67 was successfully introduced into HNTs. It can be found that the intensities of the -OH peak at 3610 cm^−1^ and 3640 cm^−1^ and the Al-OH peak at 908 cm^−1^ are weakened; it can thus be speculated that -OH provided the growth site for ZIF-67 and was covered by ZIF-67. The intensity of the Si–O–Si peak near 1031 cm^−1^ is also reduced and a blue shift can be observed on it; it can thus be speculated that the strong interaction between Co or imidazole groups in ZIF-67 and Si–O–Si in HNTs caused the successful preparation of HNTs@ZIF-67.

**Figure 6:**
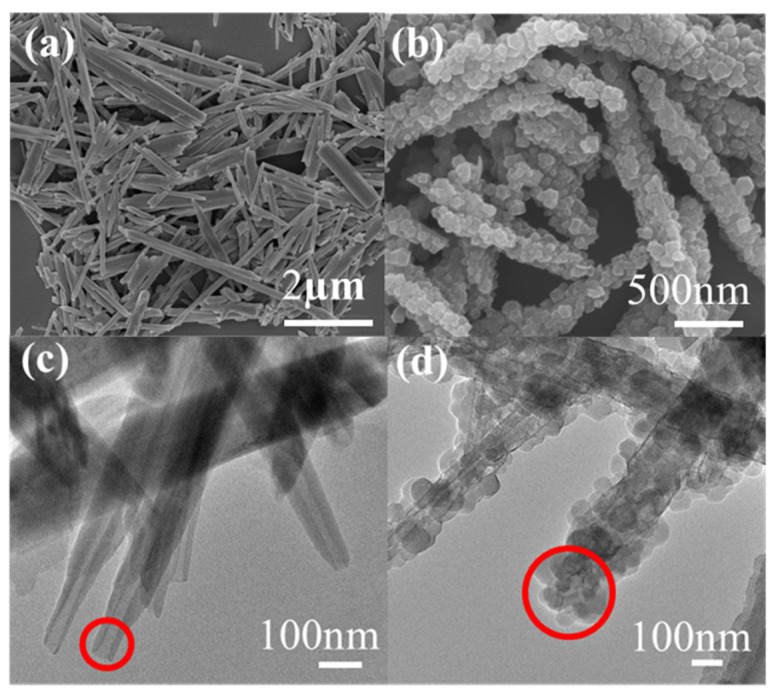
SEM and TEM images of (**a**,**c**) HNTs and (**b**,**d**) HNTs@ZIF-67.

The morphology of HNTs@ZIF-67 was observed by SEM and TEM. As shown in Figure 6a, the SEM image of unmodified HNTs indicates that the surface of these HNTs is rather smooth. Figure 6c is the TEM image of the original HNTs. It can be found that the HNTs have a hollow tubular structure and the two ends of the tube are open. The length of the tube is about 2–3 μm and the diameter is about 100 nm. Figure 6b is the SEM image of HNTs@ZIF-67. It can be seen that the surface of HNTs@ZIF-67 is rough, and the surface and both ends are coated with dense ZIF-67, and the particle size of ZIF-67 is about 50 nm. HNTs@ZIF-67 is about 3 μm in length and about 200 nm in diameter. Figure 6d is the TEM image of HNTs@ZIF-67; it can be found that the hollow structure of HNTs still exists in HNTs@ZIF-67, and ZIF-67 grew compactly, encapsulating both ends of HNTs. The above results proved that HNTs were surface-grown with both ends encapsulated by ZIF-67, confirming the successful preparation of HNTs@ZIF-67.

**Figure 7:**
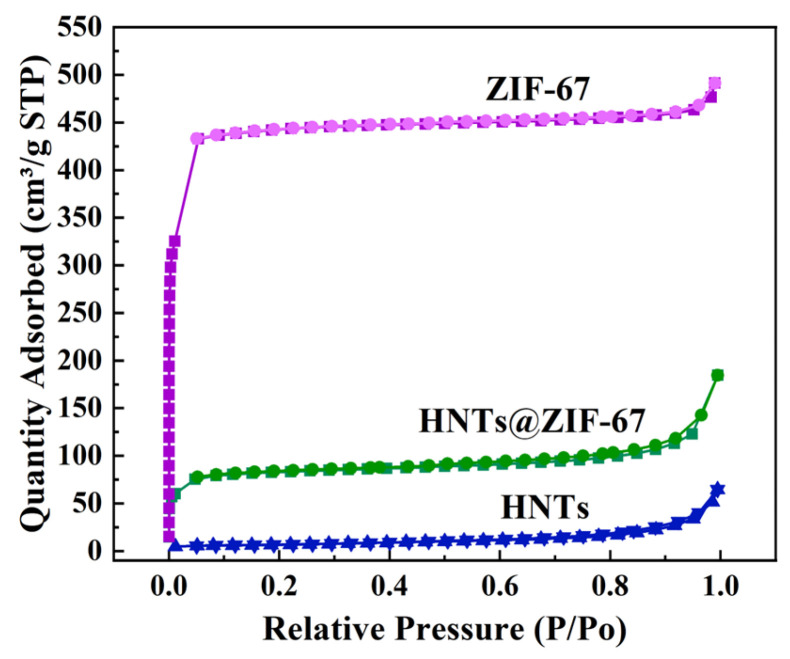
N_2_ adsorption and desorption curves of HNTs, ZIF-67 and HNTs@ZIF-67.

To explore the pore structure of HNTs, ZIF-67, and HNTs@ZIF-67, a N_2_ adsorption–desorption test was carried out. The results are shown in Figure 7. The adsorption–desorption curve of the original HNTs shows the H3 hysteresis loop and conforms to the type IV isotherm, indicating that the HNTs have a mesoporous structure [29]. The adsorption–desorption curve of the original ZIF-67 shows the type I isotherm, indicating that the ZIF-67 has a microporous structure [30]. The adsorption and desorption curve of HNTs@ZIF-67 showed an H3 hysteresis loop composite I/IV isotherm. The N_2_ adsorption–desorption process of HNTs@ZIF-67 is mainly divided into three stages: *P*/*P*o < 0.02, 0.02 < *P*/*P*o < 0.85, and *P*/*P*o > 0.85. When *P*/*P*o < 0.02, the N_2_ adsorption capacity of HNTs@ZIF-67 began to increase, indicating that ZIF-67 had a microporous structure. When *P*/*P*o > 0.85, the N_2_ adsorption capacity also increased sharply, and an obvious hysteresis loop appeared, which may be because of the mesoporous structure of HNTs. The above results indicate that HNTs@ZIF-67 is successfully prepared and constitutes a composite material with both microporous and mesoporous structures.

Combined with SEM, TEM, and BET data, it can be inferred that HNTs@ZIF-67 exhibits a hollow mesoporous HNT structure (inner diameter of 10 nm) externally coated with a dense microporous ZIF-67 (theoretical effective pore size of 3.3 Å) structure [30,31,32], and both ends of HNTs are encapsulated by ZIF-67. Theoretically, this hollow mesoporous material features a surface modified with a dense microporous layer, which enables selective separation of He (2.6 Å) and CH_4_ (3.8 Å) through both terminal openings and surface micropores, while promoting helium diffusion via its internal hollow mesoporous channels.

**Figure 8:**
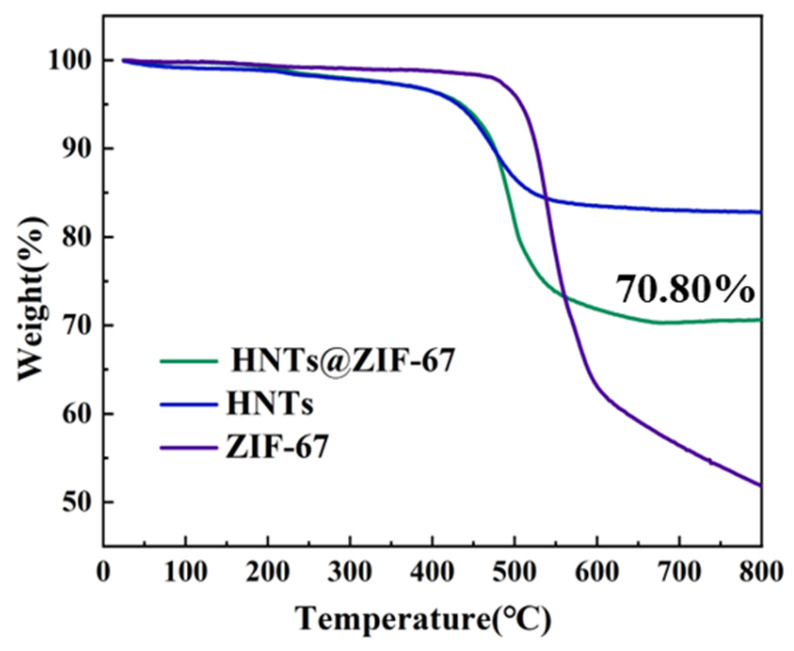
TGA curves of Composite material HNTs, ZIF-67 and HNTs@ZIF-67.

To explore the thermal stability of HNTs@ZIF-67, a thermogravimetric analyzer (TGA) was used, and the weight loss curve is shown in Figure 8. In the range of 0~200 °C, HNTs and HNTs@ZIF-67 showed a slight mass loss, which can likely be attributed to the residual water within HNTs@ZIF-67. At about 425 °C, HNTs and HNTs@ZIF-67 began to show significant mass loss, and ZIF-67 began to degrade at 500 °C. The mass loss of HNTs around 425 °C is mainly attributed to the structural degradation of HNTs [33]. The weight loss of HNTs@ZIF-67 at 425~650 °C can be attributed to the structural degradation of HNTs and the gradual pyrolysis of organic ligands of ZIF-67. HNTs@ZIF-67 has a thermal stability comparable to that of HNTs, and the initial pyrolysis temperature is about 425 °C. The residual rate of HNTs@ZIF-67 was 70.8%. According to the mass ratio of HNTs to ZIF-67 in the experiment, the theoretical residual [34,35] of HNTs@ZIF-67 was calculated according to Equation (S1), and the calculated results are listed in Table 1. It can be found that the experimental residual mass is slightly larger than the theoretical residual mass. Indicating that the interaction between HNTs and ZIF-67 improved the stability of the HNTs@ZIF-67, once again indicating that ZIF-67 successfully grew on HNTs, indicating that the interaction between HNTs and ZIF-67 improved the stability of HNTs @ ZIF-67 within *P*/*Po* < 0.02 was reduced by about 75% compared with pure ZIF-67. This may be caused by the following two reasons. First, HNTs in HNTs@ZIF-67 have no contribution to micropore adsorption, which leads to a decrease in adsorption capacity. On the other hand, the results of the infrared test show that there is an interaction between HNTs and ZIF-67, which may lead to a decrease in the pore size of ZIF-67 on the near side of HNTs, making it difficult to adsorb N2 and leading to a decrease in adsorption capacity.

### 3.2. Characterization of HNTs@ZIF-67/6FDA-TFMB MMMs

To investigate the effect of HNTs@ZIF-67 on the structure of 6FDA-TFMB, the morphology and structure of the MMMs were characterized by SEM, XRD and FTIR.

**Figure 9:**
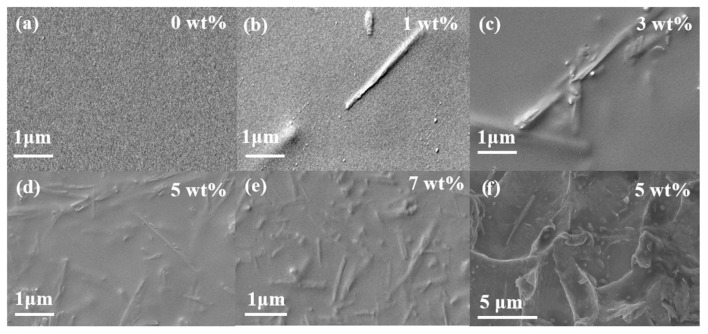
Surface and cross-section electron microscopy of different loadings: (**a**) 0 wt%, (**b**) 1 wt%, (**c**) 3 wt%, (**d**) 5 wt%, (**e**) 7 wt%, and (**f**) cross-section images of 5 wt% MMMs.

The SEM figures of the surface of pure 6FDA-TFMB and HNTs@ZIF-67/6FDA-TFMB MMMs are shown in Figure 9, the surface of pure 6FDA-TFMB is dense and nonporous, and the surface is flat and smooth. However, when the doping amount of HNTs@ZIF-67 increases, it is evident that the surface of the MMMs is gradually rough and has a large number of protrusions. Most of the protrusions are tubular, which is consistent with the morphology of HNTs@ZIF-67. Interestingly, most of the HNTs@ZIF-67 present a horizontal state distribution.

The cross-section of 6FDA-TFMB is dense and smooth, but with the addition of HNTs@ZIF-67, it exhibits a fish-scale shape, which may be caused by tensile fracture during membrane low-temperature brittle fracture, indicating that MMMs still maintain certain mechanical properties at low temperature. From the 5 wt% load profile, it can be seen that there are parts of HNTs@ZIF-67 that are vertical and inclined, and the white protrusion is horizontal. There is no obvious gap or boundary that can be observed between HNTs@ZIF-67 and the matrix, indicating that the compatibility between the filler and the matrix is better.

**Figure 10:**
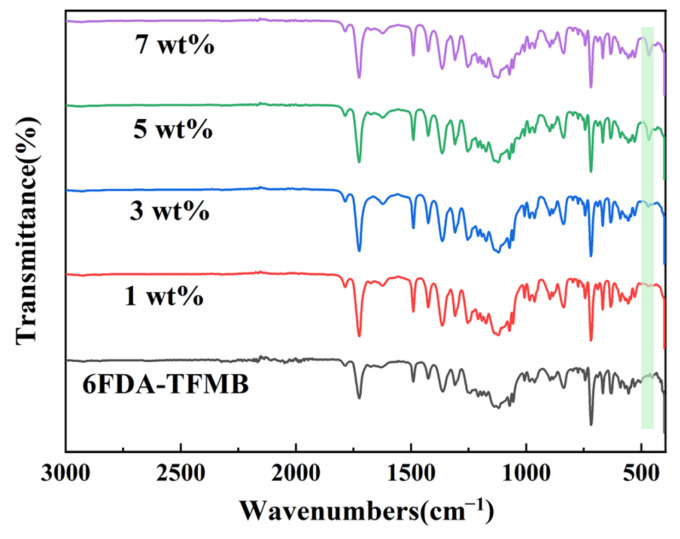
FTIR spectra of MMMs with different loadings of HNTs@ZIF-67/6FDA-TFMB.

The FTIR of HNTs@ZIF-67/6FDA-TFMB MMMs with different loadings is shown in Figure 10. The FTIR spectra of the MMMs were basically similar to those of the pure 6FDA-TFMB membranes. However, a novel absorption peak showed up at 470 cm^−1^, which is associated with the bending vibration of the Si–O bond [26], and the peak intensity increased with the increase in the loading amount of HNTs@ZIF-67, which again indicated that HNTs@ZIF-67 was successfully introduced into MMMs.

**Figure 11:**
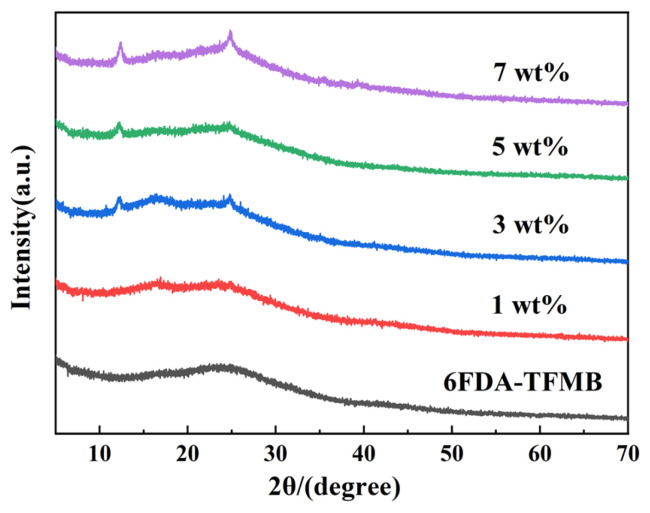
XRD spectra of MMMs with different loadings of HNTs@ZIF-67/6FDA-TFMB.

Figure 11 is the XRD data of MMMs. It can be seen that compared with pure 6FDA-TFMB, MMMs have sharp diffraction peaks at 12.36° and 25.48°, and as the filler loading increases, the intensity of the diffraction peaks becomes stronger. Compared with Figure 3, it can be found that the two peaks are the characteristic peaks of HNTs@ZIF-67, indicating HNTs@ZIF-67 has been successfully introduced into 6FDA-TFMB.

**Figure 12:**
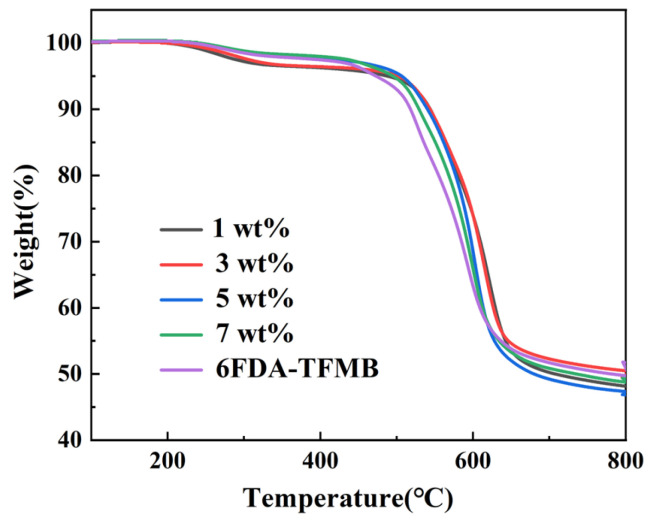
TGA result of MMMs with different loadings of HNTs@ZIF-67/6FDA-TFMB.

In industrial production, membrane separation of helium and methane from natural gas is usually carried out in the range of 20–60 °C. Thermal stability is one of the important factors affecting the service life of gas separation membranes. The increase in temperature will bring higher kinetic energy to the gas and improve the gas diffusion rate, but it will also increase the operating costs required for heating. However, it also challenges the thermal stability of the polymer membrane. The thermal stability of MMMs was tested by TGA. The mass loss of MMMs from room temperature to 800 °C in N_2_ atmosphere is shown in Figure 12. It can be seen from Figure 12 that the mass loss of 6FDA-TFMB is mainly divided into three stages: the first stage is between 120 °C and 520 °C, which is mainly due to the volatilization of residual organic solvents. The second stage is 520 °C to 640 °C, during which 6FDA-TFMB began to degrade, the carbonyl group on the imine ring began to break, and the main chain began to aromatize. The third stage is between 640 °C and 800 °C, which can be attributed to the further degradation of the defective aromatic carbon. Compared with 6FDA-TFMB and MMMs, the degradation stage of MMMs is similar to that of 6FDA-TFMB. The structure of MMMs began to pyrolyze after 500 °C, which proved that MMMs had excellent thermal stability and met the needs of most industrial production scenarios.

### 3.3. Gas Separation Performance of HNTs@ZIF-67/6FDA-TFMB MMMs

**Figure 13:**
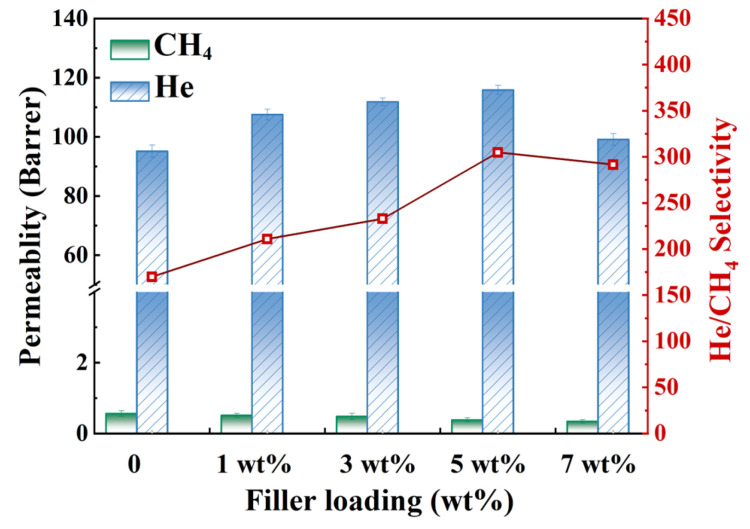
The gas separation performance diagram of MMMs with different loadings of HNTs@ZIF-67/6FDA-TFMB.

In this work, high purity He and CH_4_ were utilized as test gases to explore the gas permeability of MMMs. The gas permeability test of MMMs with different doping amounts was carried out at 35 °C and 0.2 MPa. It can be seen from Figure 13 that the introduction of HNTs@ZIF-67 makes the He permeability of the MMMs increase first and then decrease, and the selectivity and He permeability show the same trend. When the loading of the HNTs@ZIF-67 composite was 5 wt%, the permeability of He reached about 116 Barrer, which was 22.11% higher than that of the 6FDA-TFMB membrane, and the selectivity of He/CH_4_ reached about 305, which was 79.41% higher than that of the 6FDA-TFMB membrane. As the doping amount continues to increase when the loading amount reaches 7%, some fillers begin to stack, resulting in uneven dispersion of the filler, making it difficult for the hollow structure to effectively promote He diffusion in the membrane, and finally causing a decrease in He/CH_4_ selectivity.

**Figure 14:**
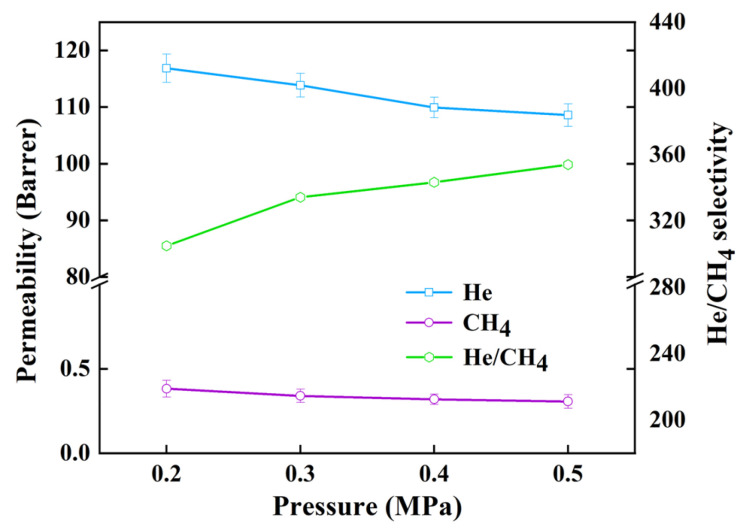
The gas separation performance results of MMMs under different pressures.

According to the above gas permeability results, when the doping amount of HNTs@ZIF-67 is 5 wt%, MMMs have the most excellent gas separation performance. Therefore, the MMMs under a doping amount of 5 wt% were chosen to explore the effect of pressure on their performance. As shown in Figure 14, with the pressure increases, there is a gradual reduction in the permeability of He and CH_4_, accompanied by a gradual increase in selectivity. The cause of this phenomenon is that the increase in pressure reduces the distance between the polymer chains and between polymer chains and fillers, resulting in a denser polymer, a smaller free volume, and a decrease in permeability. However, the kinetic diameter of CH_4_ is larger than that of He, making it more difficult for CH_4_ to pass through the compressed membrane relative to He, resulting in the increased He/CH_4_ selectivity of MMMs.

**Figure 15:**
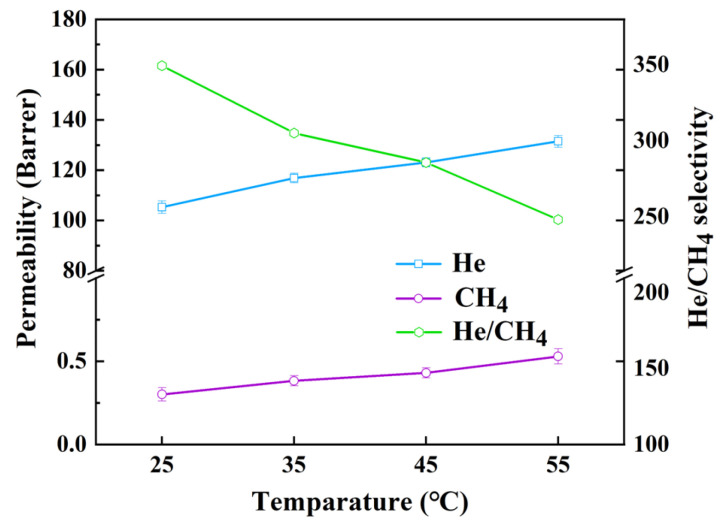
The gas separation performance results of MMMs under different temperatures.

Figure 15 shows the influence of different temperatures on the performance of MMMs. It can be seen that the permeability of He and CH_4_ increases with the increase in temperature, while the selectivity is in a downward trend. The reason is that a higher temperature gives higher potential energy to gas molecules and promotes the diffusion of gas. Meanwhile, high temperature also increases the movement of polymer chains, resulting in a decrease in mass transfer resistance. The increase in permeability of CH_4_ is larger than that of He, which ultimately leads to a decrease in selectivity. The observed higher methane permeability relative to helium can be attributed to three key factors:

(1) The difference in activation energy. According to the Arrhenius Equation (4), the higher the activation energy, the more significant the effect of temperature on the permeation rate.
  (4) $$P_{i}=Ae^{\frac{-E_{p}}{RT}}$$
  where *P_i_* is the permeability of component *i*, *A* is the pre-exponential factor, *E_P_* is the apparent activation energy (J·mol^−1^), *R* is the general gas constant (8.314 J·mol^−1^·K^−1^), and *T* is the temperature (K). The *E_P_* of He is smaller than that of CH_4_, indicating that the increase in permeability is higher when the temperature is increased.
(2) The interaction between the gas and material. He is an inert gas, and the interaction between helium and the material is weak. There is a strong adsorption between CH_4_ and the material. When the temperature increases, these interactions will be weakened, thus greatly improving the permeability of CH_4_.
(3) Differences in diffusion mechanisms. Compared with He, CH_4_ has a stronger adsorption–desorption process in diffusion. The temperature has a greater impact on the diffusion and desorption process of CH_4_. The increase in temperature may reduce the energy barrier of methane desorption, reducing the mass transfer resistance of methane and finally decreasing the selectivity of the MMMs.

### 3.4. Evaluation of Gas Separation Performance of MMMs

**Figure 16:**
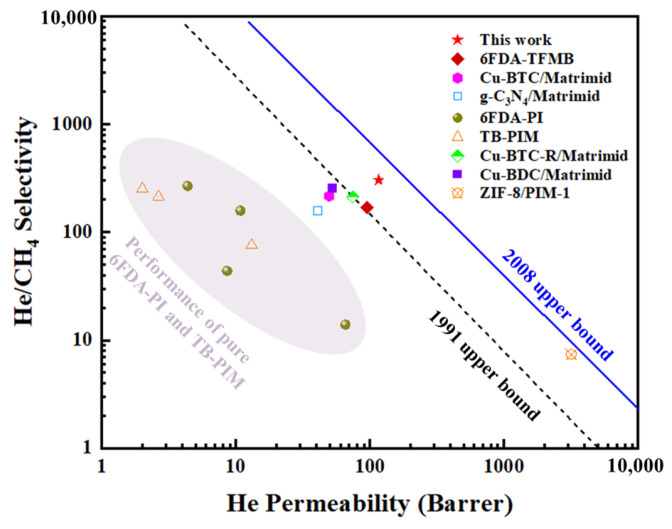
The comparison of He/CH_4_ separation performance of MMMs in this work and references.

The He/CH_4_ separation performance of HNTs@ZIF-67/6FDA-TFMB MMMs in this work was compared with that of MMMs reported in the literature over the past ten years, as summarized in Table 2. As shown in Table 2, the HNTs@ZIF-67/6FDA-TFMB mixed matrix membrane has superior helium permeability and selectivity compared with other He/CH_4_ separation membranes, and the relationship between the two is more balanced. In addition, the results of this work are compared with the 1991 Robeson upper bound and the 2008 Robeson upper bound, and the results are shown in Figure 16. The performance of HNTs@ZIF-67/6FDA-TFMB MMMs in this work breaks through the 1991 Robeson upper bound, showing certain application potential, but there is also a need for further optimization. It may be an optional optimization direction to reduce the interaction between fillers, enhance dispersion and regulate the orientation of composites to make them present a vertical state to maximize the advantages of HNTs hollow structure.

### 3.5. Mechanism Explanation of HNTs@ZIF-67/6FDA-TFMB MMMs

#### 3.5.1. Molecular Dynamics Simulation

(1) Calculation of adsorption energy

Adsorption energy is a physical quantity that describes the interaction strength between molecules, atoms, ions, and different materials and surfaces. It is used to measure the energy change and stability of the adsorption process and is often used to evaluate the adsorption capacity of materials for different gases. To further explore the adsorption relationship between gas molecules and materials, DFT was used to calculate the adsorption energy of gases on the surfaces of different materials. Molecular models of He, CH_4_, and various materials are constructed as shown in Figures S1 and S2. Taking the adsorption energy calculation of He on the ZIF-67 surface as an example, the calculation process is as follows: Firstly, the optimized He was placed at a distance of 3 Å directly above the mass center of the ZIF-67 hole, and then the DMol^3^ module was used to optimize the model. The functional was GGA-PBE, and the model was geometrically optimized to obtain the energy steady-state configuration; then the adsorption site of He on the ZIF-67 surface hole was found. Then, according to Equation (5), the binding energy between He and ZIF-67 at the energy steady-state configuration was calculated to obtain the corresponding adsorption energy. At the same time, the adsorption sites of CH_4_ on ZIF-67 and He/CH_4_ on HNTs were found, and the binding energy between them was calculated according to the above steps [39].

*E_int_* = *E_A_-_B_* − (*E_A_* + *E_B_*) (5)

where *E_int_* is the adsorption energy, *E_A-B_* is the total energy of the A-B combination, *E_A_* is the energy of individual A in the combination, *E_B_* is the energy of individual B in the combination.

The adsorption energies are summarized in Table 3. According to Table 3, it can be seen that the adsorption energies of CH_4_ gas molecules with ZIF-67, the inner and outer walls of HNTs, and 6FDA-TFMB are greater than those of He gas molecules, indicating that CH_4_ is more likely to be selectively adsorbed by HNTs@ZIF-67 and 6FDA-TFMB. At the same time, it also shows that methane desorption needs to overcome a larger energy barrier. In contrast, He is not easily adsorbed and diffuses more easily in the material. The adsorption energy between He and ZIF-67 is larger than that of the inner and outer walls of HNTs and 6FDA-TFMB, indicating that He is easily adsorbed by ZIF-67 after entering MMMs, and compared with the matrix, He tends to be transported through HNTs@ZIF-67. In summary, HNTs@ZIF-67 plays a key role in improving the He/CH_4_ performance of HNTs@ZIF-67/6FDA-TFMB MMMs.

(2) Adsorption density field

**Figure 17:**
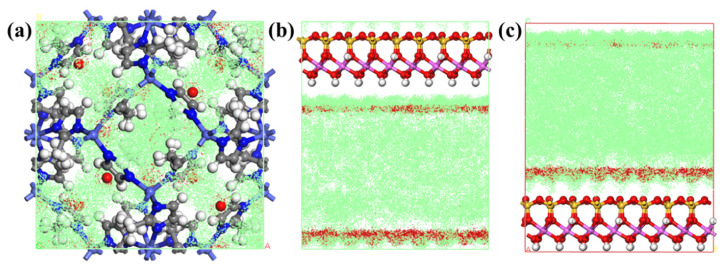
Adsorption density field of He and CH_4_ on (**a**) ZIF-67 and HNTs (**b**) inside, (**c**) outside.

Based on molecular mechanics, the adsorption density field of gas inside ZIF-67 (as shown in Figure 17a) and on the surface of HNTs (as shown in Figure 17b,c) were calculated by Grand Canonical Monte Carlo. The red points represent the CH_4_ adsorption density field, and the green points represent the He adsorption density field. It can be found from Figure 17 that CH_4_ is more likely to adsorb on the surface of HNTs inside ZIF-67, while the adsorption density field distribution of He is larger, which indicates that the adsorption energy of He by ZIF-67 and HNTs is lower. It can also be inferred that ZIF-67 and HNTs have lower diffusion barriers to He.

#### 3.5.2. Separation Mechanism of MMMs

**Figure 18:**
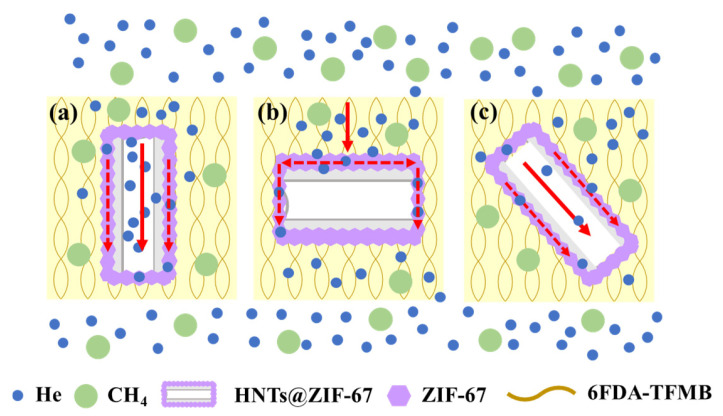
Schematic diagram of the gas permeation mechanism of HNTs@ZIF-67/6FDA-TFMB membranes. (**a**) Horizontal states, (**b**) vertical states, (**c**) inclined states.

HNTs@ZIF-67 plays an important role in improving the He/CH_4_ separation performance of MMMs. Combining the conclusions of the above experiments and simulations, the mechanism of HNTs@ZIF-67 improving the He/CH_4_ separation performance of MMMs is summarized as follows. Firstly, ZIF-67, as an important component of HNTs@ZIF-67, has an effective theoretical pore diameter of 3.3 Å, which is between that of He (2.6 Å) and CH4 (3.8 Å), giving HNTs@ZIF-67 the ability to select between He and CH_4_. HNTs, serving as a hollow support in HNTs@ZIF-67, can provide a fast mass transfer channel for gas and greatly reduce mass transfer resistance. When HNTs@ZIF-67 is doped into MMMs, the doping state of HNTs@ZIF-67 can be simply summarized as horizontal, vertical and inclined states (Figure 18). The mass transfer paths of He are depicted by the red solid line and the dashed line. In the vertical state, He has two kinds of transmission paths. (1) He can enter the hollow tube of HNTs through the ZIF-67 on the tube orifice for rapid transmission. (2) He can also perform rapid transfer in the topological structure of ZIF-67 through the continuous ZIF-67 layer on the surface of HNTs@ZIF-67. In the horizontal state, He can only be rapidly transferred in the topological structure of ZIF-67 through the continuous ZIF-67 layer on the surface of HNTs@ZIF-67. Finally, based on the above separation mechanism, the He permeability and the He/CH_4_ selectivity of MMMs were improved.

## 4. Conclusions

In this work, HNTs@ZIF-67 was synthesized using the in situ growth method, and MMMs were prepared by incorporating HNTs@ZIF-67 into 6FDA-TFMB. Then, the synthesized HNTs@ZIF-67 and MMMs were characterized, and a gas permeability test of MMMs with different HNTs@ZIF-67 loadings and at different temperatures and pressures was carried out. The results show that, with the increase in HNTs@ZIF-67 loading, the He permeability and selectivity of MMMs are enhanced. When the loading amount was 5 wt%, the He permeability and He/CH_4_ selectivity of MMMs reached 116 Barrer and 305, which were 22.11% and 79.41% higher than that of the pure 6FDA-TFMB membrane.

The calculation results of DFT and MC show that it is difficult for He to be adsorbed by ZIF-67 or HNTs compared with CH_4_, and it diffuses more easily inside ZIF-67 and on the surface of HNTs. ZIF-67 shows larger adsorption energy with He than those of HNTs and 6FDA-TFMB, indicating that He is easily adsorbed by ZIF-67 in MMMs. He exhibits lower adsorption energy with all materials compared to CH_4_, indicating that He is hard to adsorb in MMMs and diffuses more easily inside ZIF-67 and HNTs.

Based on experimental and molecular simulation results, the mechanism of HNTs@ZIF-67 improving the He/CH_4_ separation performance of MMMs was summarized. He can not only transport rapidly through HNT hollow tubes with less mass transfer resistance, but also form a continuous ZIF-67 layer on the surface of HNTs for rapid transport, which enhances the He/CH_4_ separation performance of MMMs. This work provides data and theoretical support for further research and application of composite materials similar to HNTs@ZIF-67 in the field of He separation.

## Figures and Tables

**Table 1:** Theoretical and practical residual mass of NNTs@ZIF-67.

Item	Residual Mass of HNTs@ZIF-67 (%)
Theoretical	68.92
Experimental	70.80

**Table 2:** Comparison table of HNTs@ZIF-67/6FDA-TFMB MMMs with other MMMs reported in the references in recent ten years on He/CH_4_ separation performance.

MMMs	*P*_He_ (Barrer)	*S*_He/CH4_	Literature
ZIF-8/PIM-1	3180	7.4	[35]
Cu-BTC-R/Matrimid	74.5	212.9	[36]
Cu-BTC/Matrimid	49.7	217.1	[8]
Cu-BDC/Matrimid	51.8	257.9	[8]
NH_2_-UiO-66/PMMA	~14	~1800	[37]
g-C_3_N_4_/Matrimid	40.7	160.4	[38]
HNTs@ZIF-67/6FDA-TFMB	116	305	This work

**Table 3:** Physical adsorption energy of He, CH_4_ with ZIF-67, inner and outer walls of HNTs, 6FDA-TFMB.

Gas	Binding Energy ^1^			
	ZIF-67	HNTs_outer_	HNTs_inner_	6FDA-TFMB
He	−11.12	−0.6746	−0.5949	−0.21
CH_4_	−96.12	−1.1910	−2.1341	−2.32

^1^ The binding energy unit is kcal/mol.

## Data Availability

The raw data supporting the conclusions of this article will be made available by the authors on request.

## Supplementary Materials

The following supporting information can be downloaded at: https://www.mdpi.com/article/10.3390/membranes15070197/s1, Equation (S1): The theoretical residual of HNTs@ZIF-67; Figure S1: 6FDA-TFMB polymer molecular model diagram; Figure S2: Diagrams of different molecular models: (**a**) He; (**b**) CH4; (**c**) ZIF-67; (**d**) Inner surface of HNTs; (**e**) Outer surface of HNTs; (**f**) side view of HNTs. Figure S3: The gas separation performance of pure 6FDA-TFMB and MMMs doped with 5 wt% ZIF-67 and 5 wt% HNTs@ZIF-67. Reference [34] is cited in the supplementary materials.
