# Supplementary material for: Construction of Transport Channels by HNTs@ZIF-67 Composites in a Mixed-Matrix Membrane for He/CH4 Separation
Source: Membranes (Basel). 2025 Jun 30;15(7):197. doi: 10.3390/membranes15070197 (PMC12299749; doi:10.3390/membranes15070197)
Supplement: Supplementary file 1 [file membranes-15-00197-s001.zip › membranes-3646071-supplementary.pdf]

Construction of transport channels by HNTs@ZIF-67 composites in a mixed-matrix membrane for He/CH_4 separation

Jiale Zhang[1], Huixin Dong[1], Fei Guo[1], Huijun Yi[1, 2], Xiaobin Jiang[1],Gaohong He[1] and Wu Xiao[1, *]
[1] State Key Laboratory of Fine Chemicals, Frontier Science Center for Smart Materials, Dalian University of Technology, 2 Linggong Road, Dalian, Liaoning 116024, China
[2] Beijing Yanshan Petrochemical High-Tech Company Limited, Beijing, 102500, China.
* Correspondence: wuxiao@dlut.edu.cn

1.The theoretical residual of HNTs@ZIF-67

The theoretical residual calculation of HNTs@ZIF-67 is shown in Equation (S1) [34]:

$$Mcal,(T)c = wt_{HNTs}\% \times Mexp,(T)_{HNTs} + (1- wt_{HNTs}\%) \times Mexp,(T)_{ZIF-67} \quad \text{(S1)}$$

where $wt_{HNTs}\%$ is the theoretical mass percentage of HNTs in HNTs@ZIF-67 composites, $Mexp,(T)_{HNTs}$ and $Mexp,(T)_{ZIF-67}$ are the mass loss data of HNTs and ZIF-67 obtained from the experiment, respectively.

2.Details of molecular simulation process

Materials Studio 2019 was used to complete the construction of the required model. The 6FDA-TFMB polymer matrix was obtained by imide reaction of hexafluorodianhydride and 2,2 ' -bis (trifluoromethyl) diaminobiphenyl. Firstly, according to the 6FDA-TFMB structural formula, the polymer molecular model of Figure S1 is drawn and preliminarily optimized. The force field type selected in this optimization is COMPASS II, which can reasonably reproduce the density-related properties in the polymer. Then, the 6FDA-TFMB molecular chain is constructed in the module of Build/Build Polymers-Homopolymer. By setting the force field and charge for it, and optimizing the geometry, thermodynamics and dynamics, the 6FDA-TFMB chain close to the real state can be obtained.

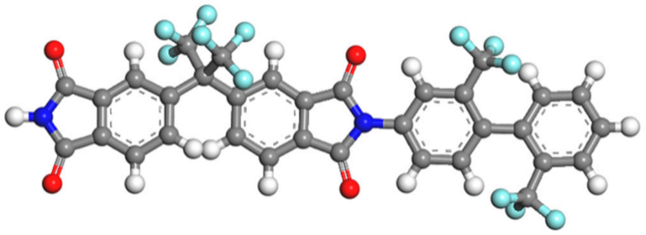

Figure S1 6FDA-TFMB polymer molecular model diagram

The corresponding molecular model of He and CH_4 can directly be drawn through the molecular structure formula. ZIF-67 and HNTs can be downloaded from the CHEMBL data, and then the force field and charge of the above molecular model are set. Then the molecular mechanics operation module Forcite is used to minimize the energy and optimize the geometric structure of the molecular model. Firstly, the geometric structure optimization of Forcite / Geometry Optimization is used to minimize the energy of the system. The universal force field is selected, and the number of optimization steps is set to 500 steps. At the same time, the

structure optimization is selected. Through this treatment, the local force in the established model system is reduced, and the unreasonable structure is eliminated, so that the model is closer to the real molecular structure of the material. The simulated 3D structures of He, CH_4, ZIF-67 and HNTs are shown in Figure S2.

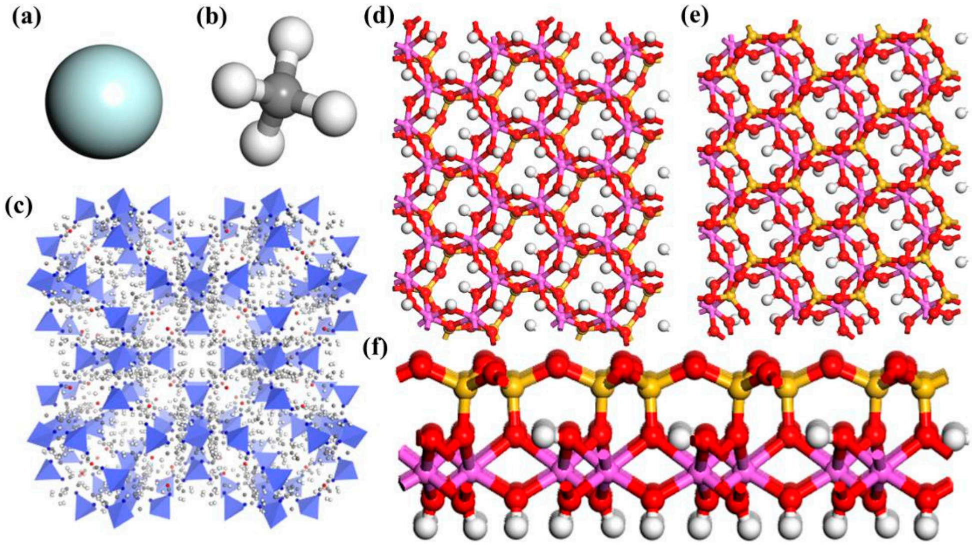

Figure S2 Diagrams of different molecular models: (a)He; (b)CH_4; (c)ZIF-67; (d)Inner surface of HNTs, (e)Outer surface of HNTs; (f)Side view of HNTs

3. Details of test error of Gas permeability

In this work, the test error calculation details of gas permeability tests refer to "GB/T 40260-2021 test method for gas permeability of polymer membrane materials". In this work, the thickness of the test membrane is controlled at about 70 μm by controlling the volume of the casting solution, and the thickness measurement process of each film is based on the "GB/T 6672-2001 Plastic membrane and sheet thickness determination mechanical measurement method". When measuring the thickness of membrane, after removing the uneven edge of the membrane, the arithmetic average of the measurement results at more than 10 different positions is taken as the thickness of the membrane.

During the gas permeability test, three test points were taken for each membrane for testing. Each test was measured at least three times under the same test conditions to ensure that the experimental error was within 5 %.

The gas permeability and selectivity of the membranes were calculated according to equations (2) and (3) in the 2.5. Gas permeability test section of the manuscript.

4. Details of test error of Gas permeability

To compare the effect of HNTs@ZIF-6 and ZIF-67 as fillers on the He/CH_4 separation performance of MMM, pure 6FDA-TFMB and MMMs doped with 5 wt% ZIF-67 and 5 wt% HNTs@ZIF-67 were tested at 0.2 MPa and 35 °C, respectively. The results are shown in Figure S3.

The He permeability of MMM loaded with 5 wt% ZIF-67 is close to pure 6FDA-TFMB, and the He/CH4 selectivity reaches about 311, which was 84.10 % higher than that of 6FDA-TFMB. While the He permeability of MMM loaded with 5 wt% HNTs@ZIF-67 increased to 116 Barrer, 22.11 % higher than that of 6FDA-TFMB, and the He/CH_4 selectivity is close to that of MMM loaded with 5 wt% ZIF-67. This indicates that HNTs@ZIF-67 can play the selective performance of ZIF-67 meanwhile constructing a continuous mass transfer channel to achieve simultaneous

improvement of He permeability and He/CH_4 selectivity.

Combining this result with the N_2 adsorption-desorption curve shown in Figure 7, although the adsorption capacity of HNTs@ZIF-67 is lower, it can still exert the selectivity performance of ZIF-67 in MMM, and even improve the He permeability. This is due to the external dense ZIF-67 layer at both ends playing a selective role in separating He and CH_4, and the special structure of HNTs@ZIF-67 allows He to transport rapidly not only in the HNTs hollow tubes but also the continuous ZIF-67 layer on the surface of HNT, finally upgrading the He/CH_4 separation performance of MMMs.

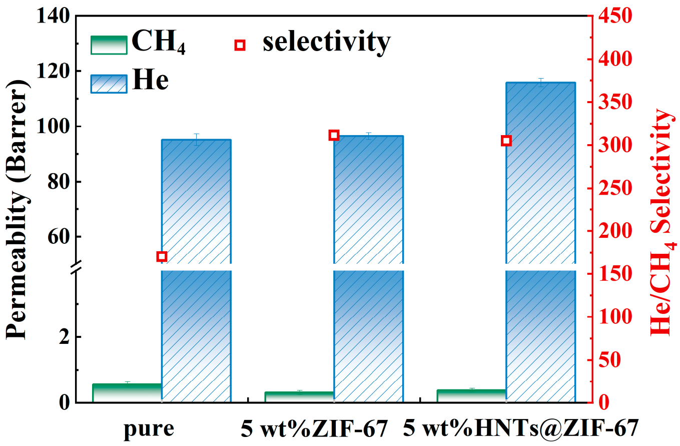

Figure S3**.** The gas separation performance of pure 6FDA-TFMB and MMMs doped with 5 wt% ZIF-67 and 5 wt% HNTs@ZIF-67.

References

[34] Dong, H.; Liu, W.; Chen, W.; Feng, T.; Wang, Y.; Piao, J.; Ren, J.; Wang, Y.; Li, S.; Chen, X.; et al. Novel C3N4/PANI@PA for enhancement of fire protection and smoke suppression in intumescent fire retardant epoxy coatings. Prog. Org. Coat. 2021, 161, 106496. https://doi.org/10.1016/j.porgcoat.2021.106496.
